# Identification of seeds based on molecular markers and secondary metabolites in *Senna obtusifolia* and *Senna occidentalis*

**DOI:** 10.1186/s40529-017-0196-4

**Published:** 2017-11-02

**Authors:** Renjun Mao, Pengguo Xia, Zhigui He, Yan Liu, Fenghua Liu, Hongguang Zhao, Ruilian Han, Zongsuo Liang

**Affiliations:** 1 State and Local Joint Research Center of TCM Fingerprint and NP Library, College of Life Sciences, Northwest A&F University, Yangling, 712100 Shaanxi China; 2 College of Life Sciences, Zhejiang Sci-Tech University, Hangzhou, 310018 Zhejiang China; 3 Tianjin Tasly Modern TCM Resources Co. Ltd., Tianjin, 300400 China; 4 Shaanxi Tasly Plants Pharmaceutical Co. Ltd., Shangluo, 726000 Shaanxi China

**Keywords:** *S. obtusifolia* seeds, *S. occidentalis* seeds, Molecular marker, ITS2 Sequence, HPLC fingerprint

## Abstract

**Background:**

*Senna obtusifolia* and *Senna occidentalis* (Leguminosae), whose seeds have similar appearance and chemical constituents, are easily confused in using their seeds. To elucidate the similarities and differences between *S. obtusifolia* seeds and *S. occidentalis* seeds, three molecular markers and high performance liquid chromatography (HPLC) were employed to evaluate the seeds characteristics of these two medicinal herbs.

**Results:**

The results showed that selected 3 ISSR and 7 SCoT primers could distinguish *S. obtusifolia* seeds from *S. occidentalis* seeds based on the specific band and UPGMA dendrogram. ITS2 sequence indicated that the intra-specific similarity of 20 *S. obtusifolia* and 16 *S. occidentalis* was 99.79 and 100.0%, respectively, while the inter-specific similarity between *S* *. obtusifolia* and *S. occidentalis* was 89.58%. Although phylogenetic analysis revealed that these two species had a close relationship, they were assigned to different branches. HPLC fingerprint results showed that seeds of *S. obtusifolia* and *S. occidentalis* shared some secondary metabolites, but aurantio-obtusin was not detected in *S. occidentalis* seeds which could differentiate *S. obtusifolia* seeds from *S. occidentalis* seeds.

**Conclusions:**

The present study not only compared the seeds characters of *S. obtusifolia* and *S. occidentalis* from molecular and secondary metabolites levels, but also provided a convenient method to identify *S. obtusifolia* seeds and *S. occidentalis* seeds effectively.

**Electronic supplementary material:**

The online version of this article (doi:10.1186/s40529-017-0196-4) contains supplementary material, which is available to authorized users.

## Background

*Senna obtusifolia* L. (Leguminosae) whose seeds known as Juemingzi in China, is a famous traditional Chinese medicine and has been regarded as a food and drug dual-purpose material by China Food and Drug Administration (CFDA). *S. obtusifolia* seeds owned various pharmaceutical properties such as eyesight improvement (Yang et al. 2012), blood lipid regulation, hypertension regulation (Li and Guo 2002) and hepatoprotective effect (Kim et al. 2009). These properties make *S. obtusifolia* seeds very popular in China and some other Asian countries. Since the wide applications in pharmaceutical and healthcare industries, the demand of *S. obtusifolia* seeds increased rapidly, and the proportion of its adulterant increased at the same time. Among the adulterants, the most common and indistinguishable one is the seeds of *Senna occidentalis* L., which is an ayurvedic medicinal herb also belong to genus *Senna* Leguminosae family. Several investigations had demonstrated that *S. occidentalis* have antibacterial (Jafri et al. 1999; Saganuwan and Gulumbe 2006), antimutagenic (Jafri et al. 1999), antiplasmodial (Tona et al. 2004) and hepatoprotective activities (Yadav et al. 2010).

Unfortunately, as the non-staple traditional medicine, the research level of *S. obtusifolia* and *S. occidentalis* is limited. The published papers about these two species mainly focus on their pharmacological characteristics (Drever et al. 2008; Ju et al. 2010) and chemical structures (Sob et al. 2008; Li et al. 2010). The authenticity of *S. obtusifolia* seeds is still identified by some traditional methods such as appearance characters or microstructures. The traditional identification methods are largely dependent on subjective experience and feelings, which easily bring confusion in identifying of *S. obtusifolia* seeds and *S. occidentalis* seeds. Even more alarming is that improper use of *S. occidentalis* seeds will cause serious symptoms (Panigrahi et al. 2014; Teles et al. 2015). However, there has no systematic research from the molecular and secondary metabolite levels to identify *S. obtusifolia* seeds and *S. occidentalis* seeds. Therefore, it is imperative to elucidate the similarities and differences between *S. obtusifolia* seeds and *S. occidentalis* seeds, establishing a suitable method to distinguish them.

In the present study, 20 *S. obtusifolia* samples and 16 *S. occidentalis* samples were collected mainly from China. ISSR and SCoT markers, ITS2 sequence and HPLC fingerprint were used to compare the differences between *S. obtusifolia* seeds and *S. occidentalis* seeds, and thereby providing an efficient method to identify them.

## Methods

### Plant materials

20 *S. obtusifolia* samples and 16 *S. occidentalis* samples were collected during 2012–2015. Detailed information of each sample had been list in Table 1. All samples were identified by Professor Yuejin Zhang (College of Life Sciences, Northwest A&F University) and voucher specimens (Specimens number see Table 1) were deposited in the State and Local Joint Research Center of TCM Fingerprint and Natural Product Library, Northwest A&F University.

**Table 1:** Detailed information of 36 samples used in the present study

No.	Name	Haplotypes	GenBank accessions	Voucher no.	No.	Name	Haplotypes	GenBank accessions	Voucher no.
1	HN-DZ-12^a^	A1	MF061316	TCMNP-1241	19	JS-TZ-14	A2	MF061317	TCMNP-1481
2	HN-ZMD-12	A3	MF085547	TCMNP-1242	20	ZJ-HZ-15	A3	MF085555	TCMNP-1571
3	HN-ZMD-13	A3	MF085550	TCMNP-1341	21	SX-YL-O13	B1	MF085530	TCMNP-0301
4	HN-ZMD-14^a^	A3	MF085548	TCMNP-1441	22	SX-YL-O14	B1	MF085531	TCMNP-0401
5	SX-YL-13	A1	MF067418	TCMNP-1301	23	SX-YL-O15	B1	MF085532	TCMNP-0501
6	SX-YL-14	A1	MF067419	TCMNP-1401	24	SX-SL-O14	B1	MF085533	TCMNP-0402
7	SX-YL-15	A1	MF067420	TCMNP-1501	25	SX-SL-O15	B1	MF085534	TCMNP-0502
8	SX-SL-15	A3	MF085549	TCMNP-1502	26	SX-LN-O14	B1	MF085535	TCMNP-0403
9	SX-WN-14	A1	MF067421	TCMNP-1402	27	HN-DZ-O14	B1	MF085536	TCMNP-0441
10	SX-LN-14	A1	MF067422	TCMNP-1503	28	YN-KM-O14	B1	MF085537	TCMNP-0461
11	SX-LN-15	A1	MF067423	TCMNP-1403	29	JS-YZ-O14	B1	MF085538	TCMNP-0481
12	Vietnam-14	A1	MF067424	TCMNP-14F1	30	JS-HA-O14	B1	MF085539	TCMNP-0482
13	Myanmar-14	A2	MF067425	TCMNP-14F2	31	GX-NN-O15	B1	MF085540	TCMNP-0551
14	SC-CD-14	A3	MF061318	TCMNP-1491	32	GX-BS-O15	B1	MF085541	TCMNP-0553
15	SD-HZ-15	A3	MF085551	TCMNP-1531	33	GX-YL-O15	B1	MF085542	TCMNP-0552
16	HB-AG-15	A3	MF085552	TCMNP-1521	34	GD-MM-O14	B1	MF085543	TCMNP-0431
17	AH-BZ-14	A3	MF085553	TCMNP-1461	35	GD-GZ-O14	B1	MF085544	TCMNP-0432
18	GX-YL-15	A3	MF085554	TCMNP-1551	36	JX-PX-O14	B1	MF085545	TCMNP-0493

HN-ZMD-14 indicated that the sample was collected from Zhumadian city, Henan province in 2014

^a^Indicated these samples were collected from wild field and the other samples were collected from the cultivars fields or experimental fields

### DNA extraction

30 sterilized seeds of each sample were placed in illumination incubator to germinate under 28 °C, 2500 lx (8 h/day) and 75% relative humidity conditions for 5 days. Young and healthy leaves from ten individuals of each sample were randomly collected and mixed for genomic DNA extraction. Total genomic DNA was extracted by using New NuClean Plant Genomic DNA Kit (CWBIO, China) according to the manufacturer’s instructions. The quality and concentration of DNA was examined by 1% agarose gel electrophoresis and spectrophotometer analysis (NanoDrop 1000, Thermo Scientific).

### PCR programs and ITS2 sequencing

ISSR and SCoT PCR reactions were conducted in 20 μL volume containing 10 μL 2 × *Taq* Mix (CWBIO, China), 8 μL ddH_2_O, 1 μL primer (1 mM), 1 μL template DNA (20 ng/μL). 100 ISSR and 85 SCoT primers were based on University of British Columbia and Collard and Mackill (Collard and Mackill 2009), respectively. All the primers were synthesized by Sangon Biological Engineering Technology & Services Company (Shanghai, China). ISSR-PCR reaction started at 94 °C for 5 min, followed by 35 cycles of 94 °C for 45 s, 55 °C for 40 s and 72 °C for 1.5 min, the final extension step at 72 °C was held for 7 min. SCoT-PCR reaction started at 94 °C for 5 min, followed by 35 cycles of 30 s at 94 °C, 1 min at 50 °C and then 1.5 min at 72 °C, with the extension at 72 °C for 7 min.

The PCR reaction for ITS2 was conducted in 50 μL volume containing 25 μL 2 × *Taq* Mix, 19 μL ddH_2_O, 2 μL primer forward (1 mM), 5′-ATGCGATACTTGGTGTGAAT-3′, 2 μL primer reverse (1 mM), 5′-GACGCTTCTCCAGACTACAAT-3′ and 2 μL template DNA (20 ng/μL). ITS2 PCR reaction started at 94 °C for 5 min, followed by 40 cycles of 94 °C for 30 s, 56 °C for 30 s and 72 °C for 45 s, the final extension step at 72 °C was held for 10 min (Chen et al. 2010). PCR reactions were performed in a Veriti 96 Thermal Cycler (Applied Biosystems, USA). PCR productions were visualized by agarose gel electrophoresis and sequenced by AuGCT Technology & Services Company (Beijing, China).

### Determination of secondary metabolites content

The secondary metabolites were determined by HPLC system (Waters, Milford, USA) equipped with a 1525 binary pump and 2487 Dual λ detector. The column was Waters Sunfire C_18_ reserved-phase column (5 μm, 250 mm × 4.6 mm). The secondary metabolites extraction and chromatography condition were according to the method described in Chinese Pharmacopoeia (Chinese Pharmacopoeia Commission 2015). Standard references of aurantio-obtusin (111900-201504), chrysophanol (110796-201520), physcion (110758-200611), emodin (110756-200110) and aloe-emodin (110795-201308) were purchased from the National Institute for the Control of Pharmaceutical and Biological Products (Beijing, China). Every sample was detected with three technical replicates to reduce the error rate.

### Data analysis

Every PCR reaction repeated twice and only unambiguous and repeatable bands were taken into account. NTSYS-pc 2.10e was employed to calculate the similarity coefficient (Rohlf 1993). The dendrograms were generated from similarity matrix data by cluster analysis using unweighted pair group method for arithmetic mean (UPGMA). The raw sequences were assembled by Codon Code Aligner V 4.0.3 (Codon Code Co., USA) based on the Hidden Markov model (HMMer) annotation method (Tamura et al. 2013). The sequence divergence and genetic distance were performed with pairwise calculations under Kimura 2-parameter model by using MEGA 6.06 software. Totally 54 samples, including 20 *S. obtusifolia*, 16 *S. occidentalis,* 15 closely related samples and three *Glycine max* samples, i.e., *Senna italica* CIMAP-C026 (Genbank Accession Number: KY492294), *Senna italica* CIMAP-C033 (KY492295), *Senna tora* CIMAP-C044 (KY492308), *Senna alexandrina* CIMAP-C032 (KY492292), *Senna alexandrina* CIMAP-C039 (KY492293), *Senna uniflora* CIMAP-C043 (KY492305), *Senna italica* (JQ301844), *Senna spectabilis* CIMAP-C027 (KY492297), *Senna spectabilis* CIMAP-C034 (KY492298), *Senna spectabilis* CIMAP-C041 (KY492299), *Senna tora* CIMAP-C037 (KY492307), *Senna tora* CIMAP-C044 (KY492308), *Senna auriculata* CIMAP-C028 (KY492300), *Senna auriculata* CIMAP-C035 (KY492301), *Senna auriculata* CIMAP-C042 (KY492302), *Glycine max* SL2 (KP793718), *Glycine max* 001 (AJ009787) and *Glycine max* Williams (KP793718), were used to construct phylogenetic tree by using Neighbor jointing (NJ) and Maximum likelihood (ML) methods, respectively.

## Results

### Discrimination ability and polymorphism of ISSR and SCoT markers

**Fig. 1:**
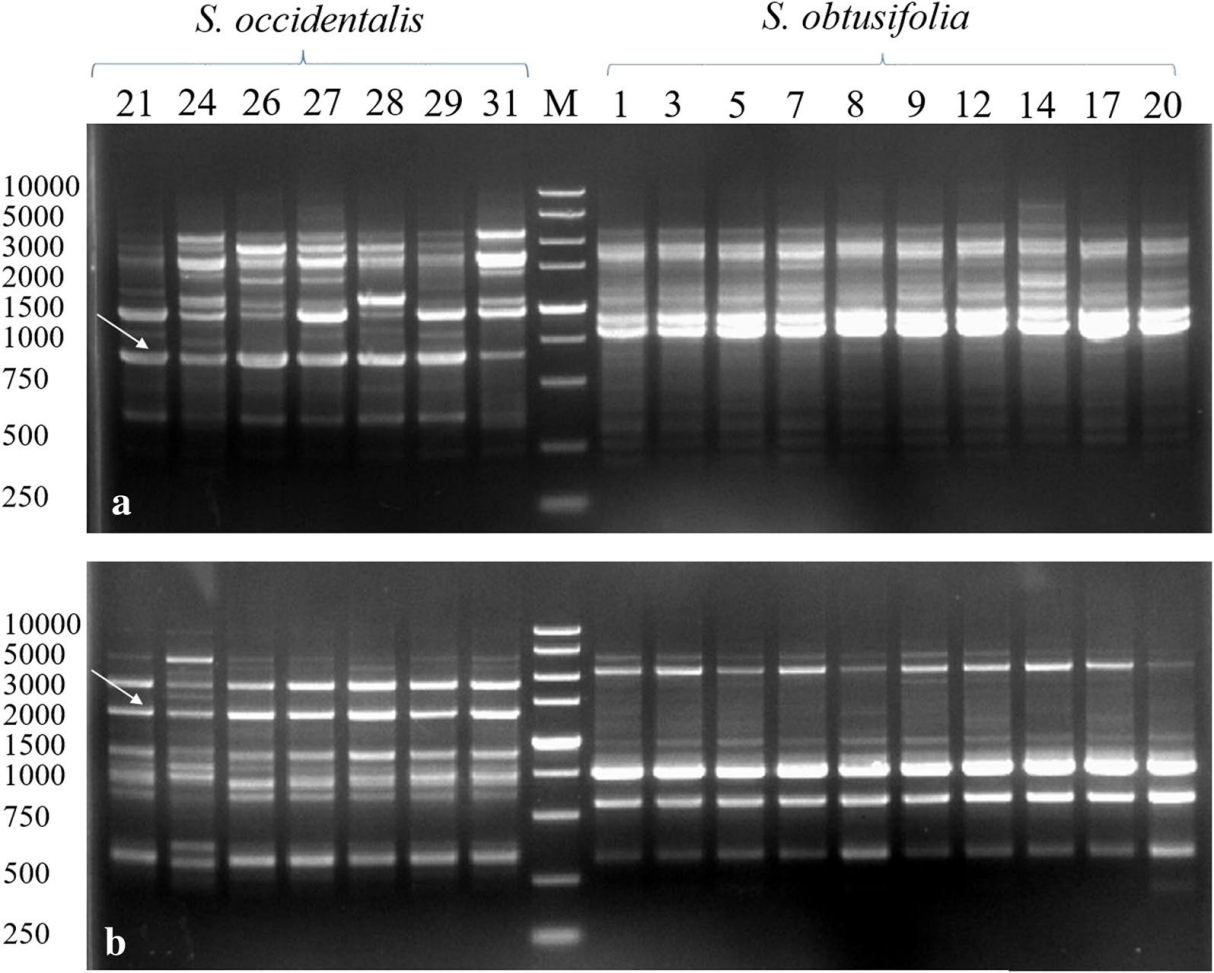
The specific band (arrow indicate) generated by primers UBC80 (**a**) and SCoT 21 (**b**). The top row numbers were same with the samples number in Table 1. 7 *S. occidentalis* samples and 10 *S. obtusifolia* samples were seperated by DL5000 Marker (TaKaRa)

Among 100 ISSR primers, 3 primers (Table 2) could generate specific band that distinguish *S. obtusifolia* from *S. occidentalis.* The primer UBC80 generated a specific band about 860 bp in *S. occidentalis* samples which did not observe in *S*. *obtusifolia* samples (Fig. 1). The primers UBC81 and UBC85 yielded the specific bands, which could distinguish *S. obtusifolia* from *S*. *occidentalis* (Additional file 1: Figure S1). 3 ISSR primers totally yielded 70 bands among 20 *S. obtusifolia* samples, 51 of which were polymorphic bands with 72.9% polymorphism and yielded 54 bands among 16 *S*. *occidentalis* samples and 35 of which were polymorphic with 64.8% polymorphism.

**Table 2:** Genetic polymorphism parameters of 36 samples generated by 3 ISSR and 7 SCoT primers

Primer	Primer sequence	Total bands		Polymorphic bands		PPB (%)	
	(5′→3′)	Sob	Soc	Sob	Soc	Sob	Soc
UBC880	(GA)8YG	29	21	20	15	68.9	71.4
UBC881	GGGT(GGGGT)2G	23	17	17	12	73.9	70.6
UBC885	BDB(CA) 7	18	16	14	8	77.8	50.0
Average	–	23.3	18.0	17.0	11.7	73.5	64.0
Total	–	70	54	51	35	72.9	64.8
SCoT 8	ACCATGGCTACCACCGCA	20	15	18	12	90.0	80.0
SCoT 21	CCATGGCTACCACCGCCT	24	17	20	14	83.3	82.4
SCoT 27	ACAATGGCTACCACTGCC	16	11	15	10	93.8	90.9
SCoT 42	ACCATGGCTACCACCGGC	18	11	14	10	77.8	90.9
SCoT 43	ACCATGGCTACCACCGCC	15	12	11	11	73.3	91.7
SCoT 44	ACGACATGGCGACCCACA	17	13	16	11	94.1	84.6
SCoT 66	ACAATGGCTACCACTAGC	21	13	18	10	85.7	76.9
Average	–	18.7	13.1	16.0	11.1	85.4	85.7
Total	–	131	92	112	78	85.5	84.8

Sob: *S. obtusifolia;* Soc: *S. occidentalis;* PPB: percentage of polymorphic bands

7 out of 85 SCoT primers could produce specific bands to distinguish *S. obtusifolia* from *S. occidentalis*. The primer SCoT21 produced a unique band about 2800 bp in *S*. *occidentalis* which could distinguish *S. obtusifolia* from *S*. *occidentalis* (Fig. 1). The other 6 SCoT primers also yielded different specific bands which could distinguish these two species (Additional file 1: Figure S1). 7 SCoT primers totally yielded 131 bands among 20 *S. obtusifolia* samples, among which 112 (85.5%) were polymorphic, and produced 92 bands among 16 *S*. *occidentalis* samples, among which 78 (84.8%) were polymorphic.

### Clustering analysis

**Fig. 2:**
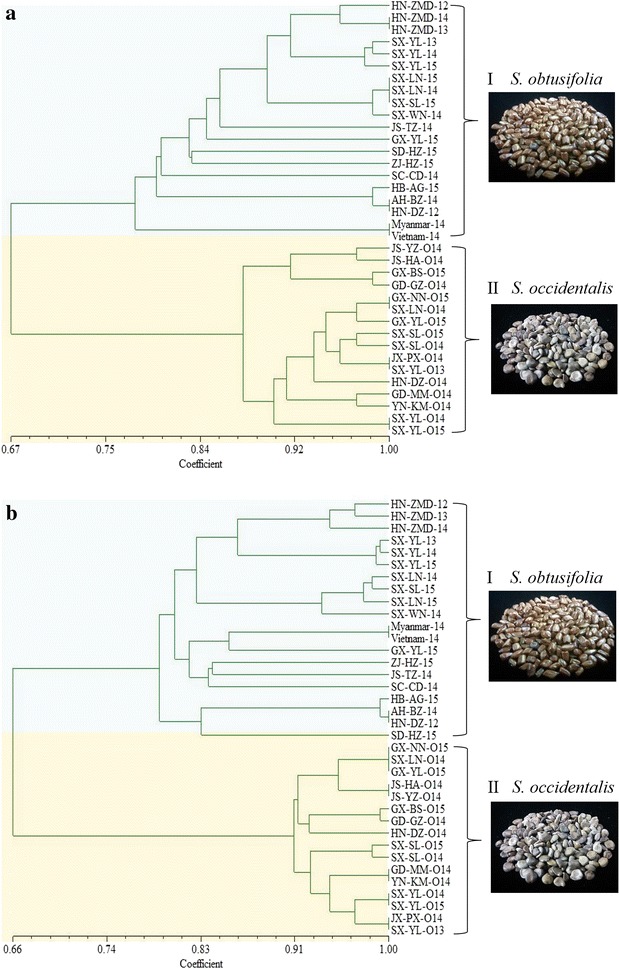
UPGMA dendrograms generated based on ISSR (**a**) and SCoT (**b**). The cluster I contained 20 *S. obtusifolia* samples and cluster II contained 16 *S. occidentalis* samples, which indicated in blue and pink respectively. The photographs of *S. obtusifolia* and *S. occidentalis* were also exhibited

The UPGMA dendrogram generated by ISSR divided 36 samples into two clusters (Fig. 2a). 20 *S*. *obtusifolia* samples and 16 *S. occidentalis* samples were assigned into cluster I and cluster II, respectively. Most samples were clustered based on their geographic origin. For example, in cluster I, the samples collected from SX-YL and HN-ZMD during 2012–2015 years were clustered together, respectively, and other four samples from Shaanxi province were nested firstly. The samples from different regions did not show a clear cluster pattern. Two samples of Myanmar-14 and Vietnam-14 were clustered and separated from samples of China. Cluster II contained all *S. occidentalis* samples. SX-LN-O14 was clustered with GX-NN-O15, and SX-YL-O13 was clustered with JX-PX-O14. The remaining samples were clustered based on their geographic regions.

The UPGMA dendrogram generated by SCoT also assigned *S*. *obtusifolia* samples and *S. occidentalis* samples to two different clusters (Fig. 2b). In cluster I, the samples from Shaanxi and Henan showed same cluster pattern with ISSR dendrogram. While the samples of Myanmar-14 and Vietnam-14 were mixed with China samples which were different from ISSR dendrogram. In cluster II, SX-LN-O14 and JX-PX-O14 were grouped with two samples from Guangxi and three samples from Shaanxi, respectively, which was not consist with their origin.

### Sequences variations and genetic distance of intra- and inter-specific

ITS2 sequences of 20 *S. obtusifolia* samples and 16 *S. occidentalis* samples were 233 bp. 20 *S. obtusifolia* samples represented 3 haplotypes namely, A1 (included 8 samples, see Table 1), A2 (2 samples) and A3 (10 samples). All 36 ITS2 of *S. obtusifolia* and *S. occidentalis* samples were submitted to GenBank and gained the accession numbers (Table 1). A1 showed the same ITS2 sequences with *Senna obtusifolia* voucher PS1588MT08 (GenBank: GQ434816). A2 and A3 showed the same sequence with voucher PH16960 (KX675057) and PH01988 (KX674796) respectively. Compared to A1, A2 showed a variation at 201 bp with ‘A’ to ‘G’ and A3 showed a variation at 216 bp with ‘A’ to ‘C’. ITS2 sequences of 20 *S. obtusifolia* samples exhibited 99.79% similarity (Additional file 2: Figure S2). Compared with the existence *S. occidentalis* ITS2 sequence (JQ301840), no variations were detected among 16 *S. occidentalis* samples (haplotypes B1). Nevertheless, compared to the high similarity value of intra-specific, the inter-specific similarity value of *S. obtusifolia* and *S. occidentalis* was 89.58% (Additional file 2: Figure S2).

The genetic distance of *S. obtusifolia* ranged from 0 to 0.01 with an average of 0.003. This value of *S. occidentalis* was 0. However, the genetic distance between *S. obtusifolia* and *S. occidentalis* ranged from 0.280 to 0.290 with the average of 0.286. The smallest genetic distance was detected between *S. obtusifolia* and *S. tora* (0.06), while the largest genetic distance was observed between *S. obtusifolia* and *Glycine max* (0.80). The results showed that the intra-specific genetic distances (0.003) was lower than the inter-specific genetic distances (0.286) between *S. obtusifolia* and *S. occidentalis*. The largest intra-specific distance was smaller than the distance of inter-specific, which could use as a parameter to distinguish *S. obtusifolia* from *S. occidentalis.*

### Phylogenetic analysis

**Fig. 3:**
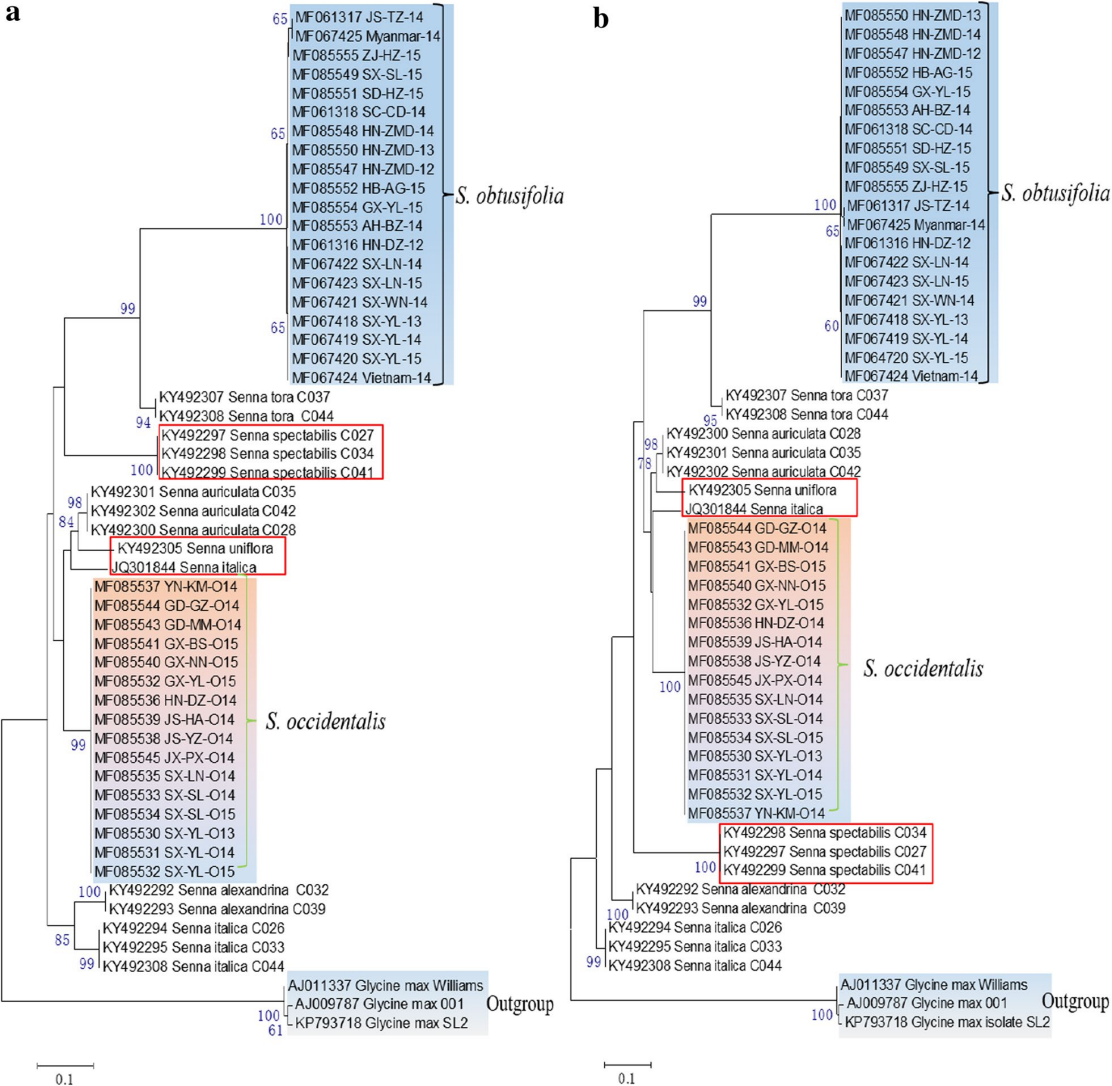
Neighbour-joining (**a**) and Maximum-likelihood (**b**) phylogenetic trees were constructed based on ITS2 sequence with 1000 replicates. The blue, pink and light blue represented *S. obtusifolia, S. occidentalis* and *Glycine max* samples, respectively. The samples differently grouped in two phylogenetic trees were highlighted in the red box

Two phylogenetic trees were constructed using Neighbor Jointing (NJ) and Maximum likelihood (ML) methods, respectively. Two phylogenetic trees showed large similarity with slightly difference (Fig. 3). 15 closely related species samples selected from GenBank were inserted into phylogenetic trees. Two *S. tora* were grouped with *S. obtusifolia* samples in both phylogenetic trees. Another difference was the branch consisted of 3 *Senna spectibilis* samples (KY492297-99) appeared a close evolution relationship with *S. obtusifolia* in NJ-tree (Fig. 3a), while it showed a relative far relationship with *S. obtusifolia* in ML-tree (Fig. 3b). *Senna uniflora* (KY492305) and *Senna italic* (JQ301844) exhibited the different positions in two trees. Despite showing some differences, both phylogenetic trees assigned *S. obtusifolia* samples and *S. occidentalis* samples to different branches (Fig. 3). Being the outgroup, three *Glycine max* samples were clustered together and separated from *Senna* samples. Collectively, the constructed phylogenetic trees could differentiate *S. obtusifolia* from *S. occidentalis* and elucidate the evolution relationship of *Senna* species.

### Determination of secondary metabolites

**Fig. 4:**
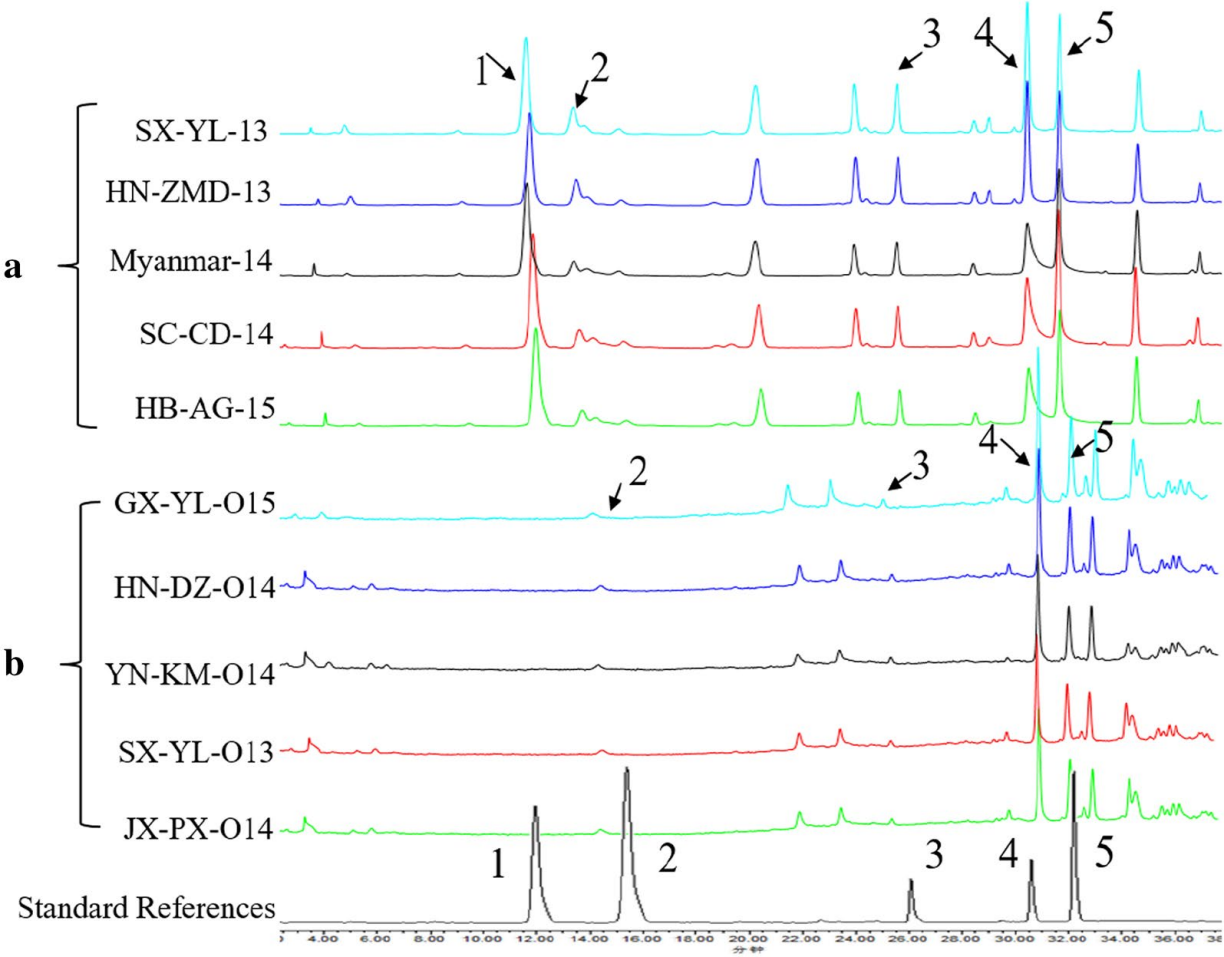
HPLC fingerprints of five *S. obtusifolia* (**a**) and *S. occidentalis* samples (**b**). Standard references from 1 to 5 represented aurantio-obtusin, emodin, aloe-emodin, chrysophanol and physcion, respectively

Representative chromatograms of each five samples of *S. obtusifolia* and *S. occidentalis* were shown in Fig. 4. The concentrations of five secondary metabolites of each sample had been listed in Additional file 3: Table S1. The HPLC fingerprints of two species showed some similarities. At the retention times of 29.33, 30.42, 33.11 and 35.23 min, these two species showed the same peaks. However, the peak detected at 12.95 min which was identified as aurantio-obtusin, was observed in *S. obtusifolia* samples only (Fig. 4). The concentrations of five secondary metabolites exhibited a large variation among different samples. The concentration of aurantio-obtusin and chrysophanol among *S*. *obtusifolia* samples ranged from 0.058 to 0.231% and 0.182 to 0.641%, respectively (Additional file 3: Table S1). Chrysophanol concentration of *S. occidentalis* seeds ranged from 0.125 to 0.315%. Physcion concentration ranged from 0.021 to 0.972 and 0.021 to 0.582% for *S. obtusifolia* and *S. occidentalis*, respectively.

## Discussion

Traditional appearance identification offered a direct method to identify medicinal herbs, but this method easily made confusion. It was more difficult to distinguish the real medicinal herb from its adulterants when they were adulterated. In addition, using one identification method only may not be suitable or convenient for all users. Molecular markers, ITS2 sequence and HPLC fingerprint were employed to evaluate the similarities and differences between *S. obtusifolia* seeds and *S. occidentalis* seeds, which could provide a suitable method to identify these two valuable medicinal herbs effectively.

Molecular markers had been widely used to identify the different genotypes of the species or the different ecological types with the same genotype. Several successful studies of plant identification were reported using different molecular marker systems. Based on selected RAPD, ISSR and SSR markers, *Jatropha curcas* samples from Mexica showed clear distinction with samples from other countries (Pamidimarri et al. 2009). Validation of the primers on *Jatropha curcas* indicated specificity of 3 RAPD primers to toxic genotypes, 5 RAPD and 2 ISSR primers to non-toxic samples and 4 RAPD primers to Mexican samples (Basha and Sujatha 2007). The study on *Olea europaea* indicated that 39 RAPD primers and 12 ISSR primers were able to distinguish 10 cultivars and 6 cultivars, respectively (Brake et al. 2014). In the present study, 3 ISSR primers and 7 SCoT primers could generate specific band to distinguish *S. obtusifolia* from *S. occidentalis*, which present a new method for identifying *S. obtusifolia*. Additionally, the results of molecular markers exhibited that both *S. obtusifolia* and *S. occidentalis* had a high level of genetic diversity and their genetic relationship showed a certain connection with geographical origin. This phenomenon could be explained by the wide cultivation area of *S. obtusifolia* and *S. occidentalis* in China for their medicinal and viewing purposes, which prompted their genetic diversity level at the same time.

The medicinal herbs collected from different regions showed large difference in quality. The adulterants exist is another reason accounting for the unstable quality of medicinal herbs. Identification of medicinal herbs based on morphological characters, microstructure or other traditional methods are lacking of the uniform standard and reliable data, which impede the further use of the medicinal herbs. Chen et al. (2010) proposed that ITS2 is a suitable DNA barcoding for medicinal plants. In recent years, ITS2 is a newly rising method which could effectively solve morphological indistinguishable or complex classification event. Additionally, ITS2 played a pivotal role in promoting the identification authenticity and stability of the medicinal herbs. ITS2 had been successively applied in identifying *Hyoscyamus niger* (Xiong et al. 2016) and *Officinal rhubarb* (Zhou et al. 2017). In this study, 36 samples of *S. obtusifolia* and *S. occidentalis* were collected from different geographic regions and evaluated their differences by ITS2. The sequence similarity of intra-specific was 99.79 and 100.0% for *S. obtusifolia* and *S. occidentalis* accordingly, while the similarity of inter-specific was 89.58%. This results demonstrated that ITS2 was a suitable DNA barcoding for identification of *S. obtusifolia*. NJ-tree separated *S. obtusifolia* and *S. occidentalis* into different branches despite that they had a close relationship. Three *Glycine max* samples formed the outgroup and separated from *Senna* species. To further evaluate the genetic relationship of *Senna* species and offer a more accurate evolution view, ML-tree was also constructed. Showing slight difference with NJ-tree, ML-tree also assigned *S. obtusifolia* and *S. occidentalis* to different branches based on ITS2 sequence. It is no doubt that using two methods to construct phylogenetic trees is helpful in ensuring the accuracy of the results and could provide more information.

Containing different kinds of secondary metabolites was a significant feature to distinguish medicinal herbs from crops or other plants. The concentration of secondary metabolite was also an indispensable parameter to evaluate the quality of the medicinal herbs. The secondary metabolite determination by HPLC can be used as a powerful tool to identify the authenticity of medicinal herbs (Tian et al. 2009; Zhang et al. 2014). In this study, the HPLC fingerprints of *S. obtusifolia* and *S. occidentalis* showed some similarities since they both belong to the genus *Senna* family leguminous. The Chinese pharmacopoeia identified Juemingzi as the dried and ripe seeds of *S. obtusifolia* or *S. tora*, not included *S. occidentalis* seeds. As a famous traditional Chinese medicine, there had already been some HPLC fingerprint reports on *S. obtusifolia* seeds (Wang et al. 2009; Huang et al. 2010). However, to our knowledge, no HPLC fingerprint research was carried out on *S. occidentalis* seeds. In this study, we presented the HPLC fingerprint of *S. occidentalis* seeds for the first time. Notably, aurantio-obtusin was not detected in *S. occidentalis* samples, which was a significant difference from *S. obtusifolia*. Former researches focus on chemical constituents also indicated that *S. occidentalis* seeds did not contain aurantio-obtusin (Yadav et al. 2010). Same to our expectation, the concentration of chrysophanol and several other anthraquinones, i.e., emodin, aloe-emodin and physcion in *S. obtusifolia* seeds were higher than that in *S. occidentalis* seeds. This results implied that *S. occidentalis* seeds maybe not suitable for medicinal use.

There is no doubt that every method has its advantages and deficiencies. Molecular marker is easy to operate and does not need expensive experimental equipment, but the number of available primers is limited. ITS2 has high accuracy and low identification error rate. However, this method requires expensive DNA sequencer and needs to further analyze after obtaining the raw sequences data, which restrict the use of this method more or less. HPLC method is affordable, accurate and could simultaneously determine the concentration of various metabolites in medicinal materials. However, the processing of the sample preparation is cumbersome and the instrument operation has a certain degree of difficulty. In this study, we presented three methods to distinguish *S. obtusifolia* from *S. occidentalis*, aiming to provide a suitable method for users from different industrials to distinguish these two species effectively.

## Conclusions

In summary, three approaches employed in this study could effectively identify *S. obtusifolia* seeds and *S. occidentalis* seeds. Selected 3 ISSR and 7 SCoT primers distinguished *S. obtusifolia* from *S. occidentalis* based on UPGMA dendrogram and agarose gel electrophoresis. ITS2 results showed that the intra-specific similarity of *S. obtusifolia* and *S. occidentalis* were 99.79 and 100.0%, respectively. While the inter-specific similarity between *S. obtusifolia* and *S. occidentalis* was 89.58%. Phylogenetic analysis assigned *S. obtusifolia* and *S. occidentalis* to different branches. HPLC fingerprints showed that two species shared some secondary metabolites, but *S. occidentalis* seeds did not contain aurantio-obtusin which could differentiate *S. obtusifolia* from *S. occidentalis*.

## Additional files

**Additional file 1: Figure S1.** The specific bands (arrow indicate) generated by ISSR and SCoT primers. The first lane numbers were same with the sample number in Table 1. 7 *S. occidentalis* samples and 10 *S. obtusifolia* samples were separated by DL5000 Marker (TaKaRa).

**Additional file 2: Figure S2.** Multiple sequences blast of ITS2 sequence of 20 *S. obtusifolia* samples (A) and 16 *S. occidentalis* samples (B) showed the intra-specific similarity was 99.79% and 100.0%, respectively. Inter-specific similarity value (C) of *S. obtusifolia* and *S. occidentalis* was 89.58%.

**Additional file 3: Table S1.** Concentrations (%) of five secondary metabolites of 20 *S. obsusifolia* samples and 16 *S. occidentalis* samples (Means±SD, n = 3).

## Abbreviations

ISSR: inter-simple sequence repeat
SCoT: target start codon polymorphism
ITS2: internal transcribed sequence 2
Sob: *Senna obtusifolia*
Soc: *Senna occidentalis*
PPB: percentage of polymorphic bands
HPLC: high performance liquid chromatography
