# Supplementary figures and images for: Identification of seeds based on molecular markers and secondary metabolites in Senna obtusifolia and Senna occidentalis
Source: Bot Stud. 2017 Nov 2;58:43. doi: 10.1186/s40529-017-0196-4 (PMC5668216; doi:10.1186/s40529-017-0196-4)

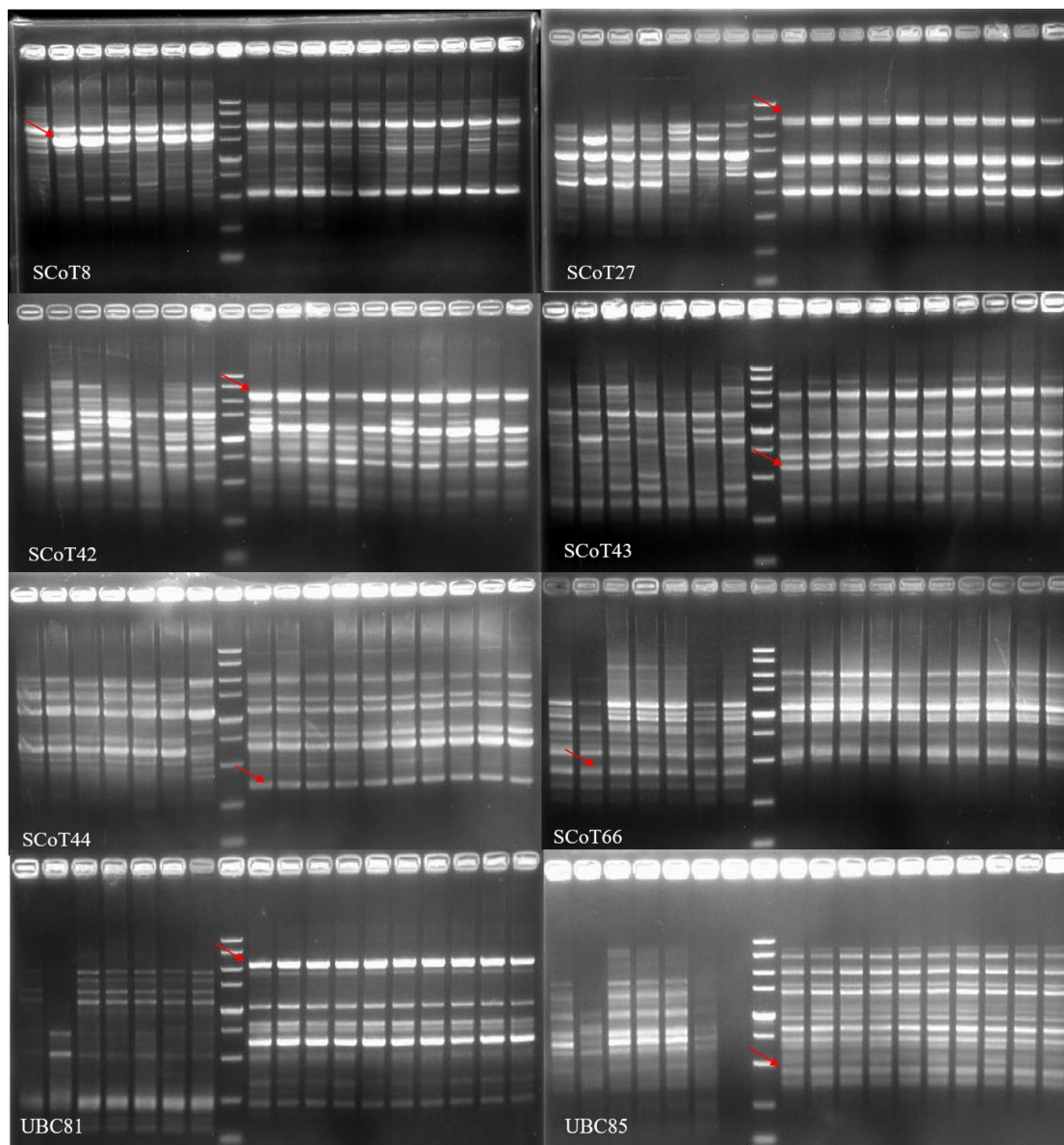

Supplement: Supplementary file 1 — Additional file 1: Figure S1. The specific bands (arrow indicate) generated by ISSR and SCoT primers. The first lane numbers were same with the sample number in Table 1. 7 S. occidentalis samples and 10 S. obtusifolia samples were separated by DL5000 Marker (TaKaRa). [file 40529_2017_196_MOESM1_ESM.pdf]

A

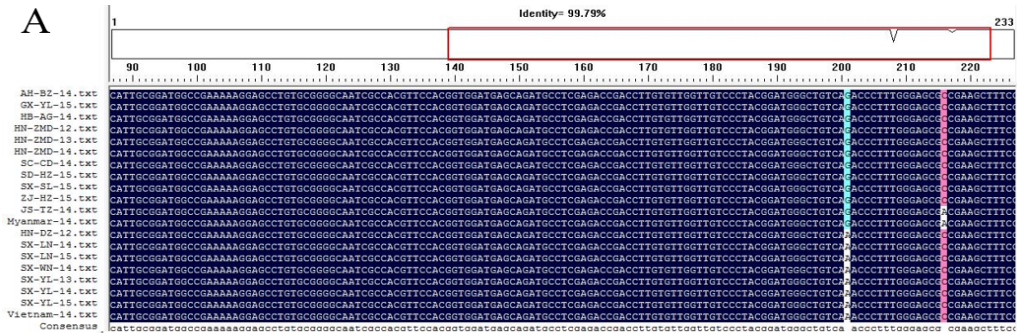

B

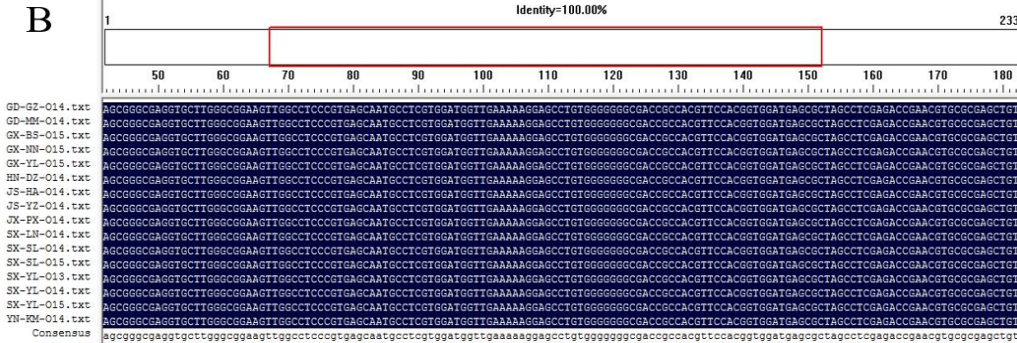

C

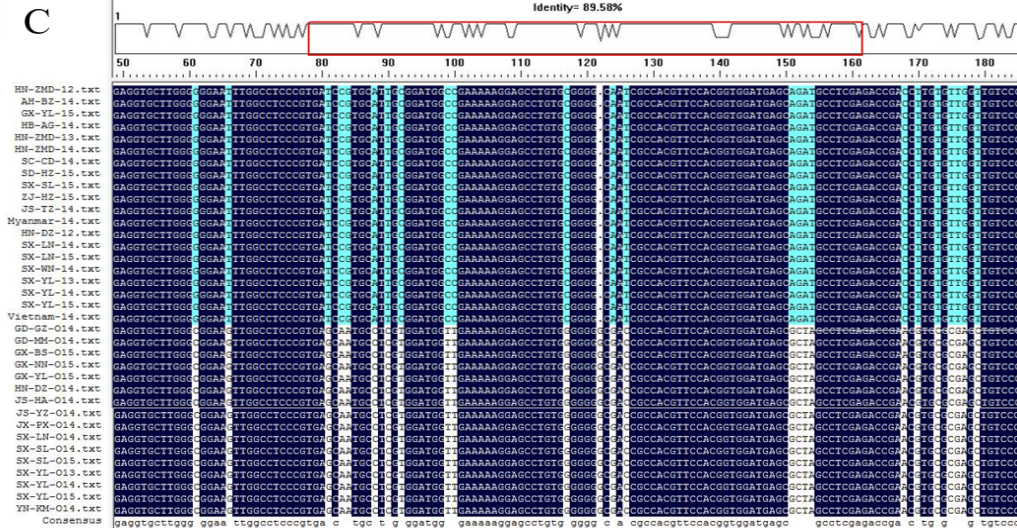

Supplement: Supplementary file 2 — Additional file 2: Figure S2. Multiple sequences blast of ITS2 sequence of 20 S. obtusifolia samples (A) and 16 S. occidentalis samples (B) showed the intra-specific similarity was 99.79% and 100.0%, respectively. Inter-specific similarity value (C) of S. obtusifolia and S. occidentalis was 89.58%. [file 40529_2017_196_MOESM2_ESM.pdf]
