# Supplementary material for: Identification of seeds based on molecular markers and secondary metabolites in Senna obtusifolia and Senna occidentalis
Source: Bot Stud. 2017 Nov 2;58:43. doi: 10.1186/s40529-017-0196-4 (PMC5668216; doi:10.1186/s40529-017-0196-4)
Supplement: Supplementary file 3 — Additional file 3: Table S1. Concentrations (%) of five secondary metabolites of 20 S. obsusifolia samples and 16 S. occidentalis samples (Means±SD, n = 3). [file 40529_2017_196_MOESM3_ESM.docx]

**Table S1.** Concentrations (%) of five secondary metabolites of 20 *S. obsusifolia* samples and 16 *S. occidentalis* samples (Means±SD, n=3).

| No. | Name | Aurantio-  obtusin | Chrysophanol | Emodin | Aloe-emodin | Physcion |
| --- | --- | --- | --- | --- | --- | --- |
| 1 | HN-DZ-12 | 0.081±0.006 | 0.215±0.012 | 0.081±0.005 | 0.081±0.006 | 0.195±0.012 |
| 2 | HN-ZMD-12 | 0.095±0.011 | 0.191±0.015 | 0.425±0.031 | 0.881±0.054 | 0.923±0.078 |
| 3 | HN-ZMD-13 | 0.085±0.012 | 0.312±0.021 | 0.805±0.066 | 0.215±0.026 | 0.128±0.024 |
| 4 | HN-ZMD-14 | 0.088±0.005 | 0.265±0.012 | 0.337±0.024 | 0.395±0.021 | 0.138±0.031 |
| 5 | SX-YL-13 | 0.082±0.004 | 0.221±0.009 | 0.688±0.037 | 0.591±0.048 | 0.281±0.022 |
| 6 | SX-YL-14 | 0.086±0.004 | 0.322±0.011 | 0.554±0.033 | 0.268±0.034 | 0.175±0.008 |
| 7 | SX-YL-15 | 0.096±0.005 | 0.182±0.010 | 0.075±0.004 | 0.091±0.011 | 0.135±0.010 |
| 8 | SX-SL-15 | 0.105±0.009 | 0.332±0.022 | 0.367±0.021 | 0.339±0.042 | 0.624±0.044 |
| 9 | SX-WN-14 | 0.092±0.006 | 0.361±0.017 | 0.635±0.041 | 0.098±0.010 | 0.035±0.003 |
| 10 | SX-LN-14 | 0.086±0.014 | 0.497±0.034 | 0.671±0.035 | 0.794±0.071 | 0.066±0.002 |
| 11 | SX-LN-15 | 0.088±0.012 | 0.641±0.066 | 0.452±0.001 | 0.855±0.082 | 0.096±0.012 |
| 12 | Vietnam-14 | 0.061±0.006 | 0.310±0.021 | 0.026±0.005 | 0.172±0.015 | 0.035±0.007 |
| 13 | Myanmar-14 | 0.058±0.004 | 0.192±0.008 | 0.017±0.001 | 0.171±0.021 | 0.021±0.004 |
| 14 | SC-CD-14 | 0.083±0.006 | 0.301±0.030 | 0.526±0.017 | 0.175±0.011 | 0.094±0.011 |
| 15 | SD-HZ-15 | 0.128±0.014 | 0.352±0.025 | 0.663±0.021 | 0.232±0.023 | 0.251±0.032 |
| 16 | HB-AG-15 | 0.182±0.046 | 0.334±0.031 | 0.511±0.023 | 0.881±0.051 | 0.821±0.091 |
| 17 | AH-BZ-14 | 0.096±0.022 | 0.291±0.020 | 0.282±0.011 | 0.215±0.013 | 0.098±0.009 |
| 18 | GX-YL-15 | 0.231±0.010 | 0.384±0.033 | 0.318±0.014 | 0.395±0.014 | 0.156±0.011 |
| 19 | JS-TZ-14 | 0.105±0.011 | 0.241±0.021 | 0.025±0.001 | 0.591±0.015 | 0.252±0.001 |
| 20 | ZJ-HZ-15 | 0.122±0.008 | 0.454±0.036 | 0.022±0.001 | 0.172±0.011 | 0.972±0.058 |
| 21 | SX-YL-O13 | — | 0.228±0.013 | 0.010±0.001 | 0.058±0.004 | 0.582±0.033 |
| 22 | SX-YL-O14 | — | 0.202±0.012 | 0.015±0.001 | 0.794±0.043 | 0.343±0.021 |
| 23 | SX-YL-O15 | — | 0.269±0.014 | 0.018±0.002 | 0.175±0.005 | 0.133±0.008 |
| 24 | SX-SL-O14 | — | 0.235±0.012 | 0.025±0.004 | 0.105±0.001 | 0.101±0.012 |
| 25 | SX-SL-O15 | — | 0.284±0.011 | 0.006±0.001 | 0.044±0.001 | 0.136±0.014 |
| 26 | SX-LN-O14 | — | 0.276±0.015 | 0.013±0.001 | 0.125±0.003 | 0.956±0.085 |
| 27 | HN-DZ-O14 | — | 0.208±0.009 | 0.008±0.001 | 0.035±0.002 | 0.051±0.001 |
| 28 | YN-KM-O14 | — | 0.315±0.021 | 0.018±0.001 | 0.026±0.002 | 0.056±0.005 |
| 29 | JS-YZ-O14 | — | 0.219±0.011 | 0.025±0.003 | 0.018±0.001 | 0.078±0.005 |
| 30 | JS-HA-O14 | — | 0.026±0.012 | 0.022±0.002 | 0.053±0.003 | 0.035±0.022 |
| 31 | GX-NN-O15 | — | 0.301±0.015 | 0.014±0.001 | 0.024±0.001 | 0.086±0.008 |
| 32 | GX-BS-O15 | — | 0.282±0.005 | 0.015±0.002 | 0.416±0.033 | 0.096±0.008 |
| 33 | GX-YL-O15 | — | 0.135±0.012 | 0.012±0.001 | 0.040±0.011 | 0.021±0.001 |
| 34 | GD-MM-O14 | — | 0.125±0.011 | 0.011±0.001 | 0.068±0.005 | 0.036±0.002 |
| 35 | GD-GZ-O14 | — | 0.202±0.008 | 0.022±0.003 | 0.662±0.0016 | 0.066±0.003 |
| 36 | JX-PX-O14 | — | 0.148±0.008 | 0.026±0.004 | 0.098±0.006 | 0.034±0.002 |

—: Not detected
